# Anti-cancer properties of *Caulerpa racemosa* by altering expression of Bcl-2, BAX, cleaved caspase 3 and apoptosis in HeLa cancer cell culture

**DOI:** 10.3389/fonc.2022.964816

**Published:** 2022-09-20

**Authors:** Happy Kurnia Permatasari, Defny Silvia Wewengkang, Nur Iedha Tertiana, Farida Zharfani Muslim, Muhammad Yusuf, Shintya Octaviana Baliulina, Vanessa Pradna Adyana Daud, Aurielle Annalicia Setiawan, Fahrul Nurkolis

**Affiliations:** ^1^ Biochemistry and Biomolecular, Faculty of Medicine, Brawijaya University, Malang, Indonesia; ^2^ Pharmacy, Faculty of Mathematics and Sciences, Sam Ratulangi University, Manado, Indonesia; ^3^ Medical School, Faculty of Medicine, UIN Maulana Malik Ibrahim Malang, Malang, Indonesia; ^4^ Medical Programme, Faculty of Medicine Universitas Brawijaya, Malang, Indonesia; ^5^ Biological Sciences, State Islamic University of Sunan Kalijaga (UIN Sunan Kalijaga), Yogyakarta, Indonesia

**Keywords:** anti-cancer, anti-proliferative, cervical cancer, HeLa cell, apoptosis, Bcl-2, viability, cleaved-caspase 3

## Abstract

The main cause of cervical cancer is infection with Human Papilloma Virus (HPV). Loss of apoptotic control allows cancer cells to survive longer and allows time for mutation accumulation thereby increasing the ability to invade during tumor development. Treatment options for cervical cancer today are surgery, radiotherapy, and chemotherapy. Toxicity to normal cells, adverse side effects, and drug resistance are the main barriers to the use of chemotherapy. Among marine organisms such as bacteria, fungi, actinobacteria, and seaweed have been used for the treatment of cancer. *Caulerpa* has bioactive metabolites, namely alkaloids, terpenoids, flavonoids, steroids and tannins and its bioactivity has been reported against many diseases including cancer. This study aimed to evaluate the anticancer activity of *C. racemosa* on *HeLa* cervical cancer cells. The study used a true experimental post-test only control group design to determine the effect of *C. racemosa* extract on *HeLa* cancer cells. *C. racemosa* extract was given in doses of 50 μg/mL, 100 μg/mL, 200 μg/mL, and 0 μg/mL as controls. Quantitative measurement of apoptosis was measured using flowcytometry and the expression of Bcl-2, BAX, and cleaved-caspase 3 as pro and anti-apoptotic proteins was measured using immunofluorescence. Trypan blue exclusion test was performed to measure cell viability. *C. racemosa* extract significantly increased the expression of pro-apoptotic proteins BAX and cleaved caspase-3 compared to controls. Annexin V-PI analysis showed the induction of apoptosis in treated cells and decreased *HeLa* cell viability at 24 hours and 48 hours post-treatment (p-value <0.05). *C. racemosa* extract has potential as an anti-cancer with pro-apoptotic and anti-proliferative activity on *HeLa* cancer cells and can be explored further as a cervical cancer therapy.

## Introduction

Cancer is a major cause of morbidity and mortality worldwide (1). Data from the Global Cancer Observatory 2020 from the World Health Organization (WHO) provides an overview of cervical cancer cases as the second most common type of cancer in women in Indonesia as many as 36,633 cases or 17.2% of the total cases (2). The main risk factor for the development of preinvasive or invasive cervical carcinoma is HPV infection. HPV infection is common, especially in young women. Persistence of HPV infection leads to an increased risk of developing precancerous and cancerous lesions. Subtypes 16 and 18 are the types most closely associated with dysplasia and advanced cancer. Acute infection with HPV types 16 and 18 confers an 11-fold to 16.9-fold risk of rapid progression of high-grade Cervical Intraepital Neoplasia (CIN) (3).

After infection, the human papillomavirus encodes proteins E6 and E7 which together promote cell proliferation, prolong cell cycle progression, and prevent apoptosis (4, 5). E6 and E7 initiate oncogenesis through interactions with tumor suppressor genes—*TP53* for E6 and retinoblastoma protein for E7 (6, 7). *TP53* has an important role in protecting genome integrity through apoptosis or inducing cell cycle arrest until errors in DNA replication can be corrected (8). E6 targets *TP53* for degradation *via* the ubiquitin pathway, preventing apoptosis and allowing potentially transformed cells to replicate (7, 9). E7 contributes to oncogenesis through its interaction with members of the retinoblastoma family RB1, RBL1, and RBL2, termed pocket proteins. E7 binds to this protein and targets it for degradation. This action results in the release and activation of the transcription factor E2F that drives the expression of S-phase genes, including the genes encoding cyclins A and E, which in turn accelerates cell cycle entry and promotes DNA synthesis (10–12).

Apoptosis is a regulated program for cell death (13). *TP53* located on chromosome 17pl3 is involved in cell proliferation, cell cycle regulation, DNA repair, promoting apoptosis, suppressing angiogenesis and tumor cell migration thereby preventing metastasis. Inactivation and malfunction of the *p53* gene have been found to be associated with the development and progression of human cancers including cervical cancer. The onco-protein of the HPV virus is known to regulate host tumor suppressor proteins such as *p53* leading to dysfunction of tumor suppressor proteins (14–18). The *p53* protein regulates the expression of Bcl-2 and BAX which play a role in apoptosis. *P53* degradation decreases tumor suppression and regulation of caspase-dependent pathways (19, 20). In the cytoplasm, cytochrome c interacts with APAF 1, procaspase-9, and ATP to form multiprotein complexes called apoptosomes. As a result of this interaction, procaspase-9 is converted into its active form, then acts on procaspase-3 and forms caspase-3. Loss of apoptotic control allows cancer cells to survive longer and allows time for accumulation of mutations that can enhance invasiveness during tumor development (20, 21).

Treatment options for cervical cancer today are surgery, radiotherapy, and chemotherapy. The three therapies can be combined (22). The failure rate of chemotherapy in solid tumors over the past six decades is 90% according to government agencies and industry (23). Toxicity to normal cells, adverse side effects, and drug resistance are major barriers to chemotherapy use (24). Because chemotherapy drugs circulate in the blood, side effects are widespread in the body including nausea and vomiting, loss of appetite, hair loss, dry mouth, fatigue, diarrhea, anemia, neutropenia, and thrombocytopenia (22). This effect is considered to reduce the patient’s quality of life (24).

**Figure 1:**
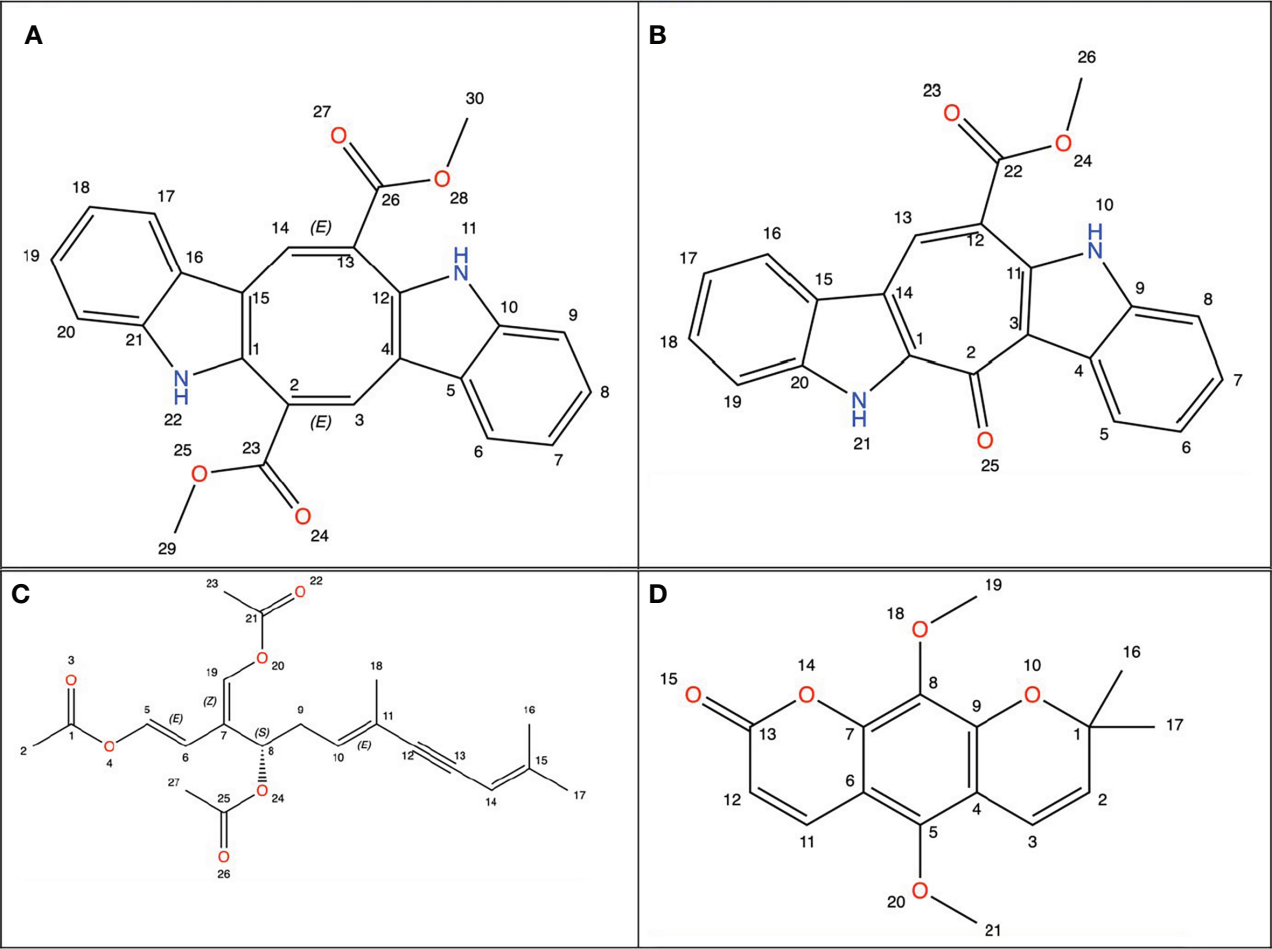
Chemical structure from *Caulerpa’s* anticancer metabolites. **(A)** Caulerpin; **(B)** Caulersin; **(C)** Caulerpenyne; **(D)** Racemosin.

Studies to find new therapeutic agents with better pharmaco-toxicological profiles are still ongoing (25). Natural marine products have been used as compounds for drug discovery (26). Among marine organisms such as bacteria, fungi, actinobacteria, and seaweed have been used for the treatment of cancer (27). Sea grapes is a term for varieties of green seaweed of the genus *Caulerpa* (28). *Caulerpa* was found to have very high economic value (29). *Caulerpa* species have been recorded in the FAO data set as one of the fundamental algae for business use particularly as nourishment for human being (30). They are frequently utilized in plated of mixed greens and vegetables, beauty care products, and drugs (31–33). Japanese people known to begin consuming sea grapes one of them as medication (antidiabetic and rheumatism). In Indonesia sea grapes usually consumed as vegetables (29, 34). *Caulerpa* has bioactive metabolites, namely alkaloids, terpenoids, flavonoids, steroids and tannins and its bioactivity has been reported against many diseases including cancer. *Caulerpa*’s anticancer metabolites such as caulerpenyne (Cyn), caulerpin, caulersin, and racemosin C, have unique structural features and are known to exhibit different effects on cancer cells (35), whose chemical structure is depicted below using ChemDraw visualization (**Figure 1**) (ChemDraw 21.0.0).

There has been no research on the benefits of *C. racemosa* originating from Indonesia. In addition, the mechanism of apoptosis signaling that become the target of *C. racemosa* is not yet clear. Therefore, based on this background, it is necessary to study the potential of *C. racemosa* from Indonesia as an anticancer by inhibiting antiapoptosis (Bcl-2), increasing proapoptosis (BAX) and *cleaved caspase-3 in vitro*. To determine the expression of Bcl-2, BAX, and caspase 3, immunofluorescence method was used. While to determine the ability of apoptosis this study using flowcytometry and trypan blue exclusion test.

## Materials and methods

### HeLa cell culture

*HeLa* cancer cells were obtained from the American Type Culture Collection (ATCC) and stored in the Biomedical Laboratory, Faculty of Medicine, Universitas Brawijaya, Malang, Indonesia. The cells were then incubated at 37 °C in a 5 percent CO_2_ incubator. *HeLa* cells were cultured in media supplemented with Dulbecco’s Modified Eagle Medium (DMEM) containing 10% fetal bovine serum (FBS) and antibiotics (100 l/ml-penicillin, 100 l/ml-streptomycin) and kept at pH 7.2-7.4. Cells were routinely grown and harvested using a trypsin-EDTA solution (36). Under a microscope, *HeLa* cell cultures were treated until they reach of 80% confluence.

### *Caulerpa racemosa* extract

*Caulerpa racemosa* was found in shallow seawater (5-10 meters above sea level) in Mantehage, North Sulawesi, Indonesia. Botanical identification and authentication were confirmed at the Department of Pharmacology, Faculty of Mathematics and Natural Sciences, Sam Ratulangi University, Indonesia. The extraction was started at Sam Ratulangi University and finished at the Biomedical Laboratory, Faculty of Medicine, Universitas Brawijaya. The extract was macerated in 96 percent ethanol for 72 hours. The total filtrate was concentrated and evaporated at 40°C to obtain a thick extract, and DMSO was used as a solvent.

### Trypan blue exclusion assay

*HeLa* cells were grown in 24-well plates and exposed to *C. racemosa* extract at various concentrations, including 0 μg/mL, 50 μg/mL, 100 μg/mL, and 200 μg/mL. The principle underlying this method is that living cells have cell membranes. Dead cells are not intact. As a result, the color will be absorbed by the dead cells (37). Trypsinization was accomplished by infusing 250 L of 0.25 percent Trypsin-EDTA for 10 minutes. The cells were suspended, and 20 L of water was taken. Viability was determined using a hemocytometer after staining with trypan blue and 10 L of cell suspension (38, 39). At 24 and 48 hours, viability was assessed.

### Immunofluorescence assay for cell apoptosis

Method adapted from Permatasari et al., 2021; Sorrells et al., 2013; Bressenot et al., 2009. Cell cultures were placed in a 12-well plate with an 18 mm diameter circular glass cover on the bottom of each well (Matsunami, Japan). Cells were washed twice in PBS before being fixed in 4 percent formaldehyde in PBS for 15 minutes at room temperature. The cells were then rinsed twice more with PBS. Permeabilization was achieved by incubating cells in PBS at 4°C for 10 minutes with 2 ml 0.1-0.5 percent Triton X-100. The cells were washed three times with PBS after being aspirated with Triton X-100. For 1 hour, cells were blocked in blocking buffer (10% goat serum, 2% bovine serum albumin, 0.2 percent Triton-X) (32, 40, 41).

Primary antibodies, namely mouse BAX primary antibody (Bioss) and rabbit Bcl-2 primary antibody (Bioss), were used to evaluate Bcl-2 and BAX expression, followed by secondary antibodies, namely anti mouse FITC (abcam) and anti-rabbit rhodamine (abcam) labeled with fluorochrome. Meanwhile, the expression of cleaved caspase-3 was evaluated using a rabbit cleaved caspase-3 primary antibody (CST, 9661S) followed by an anti-rabbit IgG Alexa Fluor-488 (abcam) fluochrome-labeled secondary antibody. The antibodies were diluted in blocking solution before being incubated in the dark at 4°C overnight. The samples were then washed five times in PBS. The nucleus was stained with 4,6-diamino-2-phenylindole (DAPI; Sigma Aldrich, Missouri, USA) at a concentration of 1 g/ml for 15 minutes before being washed with PBS six times. Following that, they were provided. They were then given mounting material and inspected with a 200x magnification Olympus IX71 inverted fluorescent microscope. The intensity of the result from immunofluoresence was quantified using ImageJ software (42, 43)

### Annexin-V/PI staining

Flow cytometry was used to determine the extent of cell apoptosis using the Annexin V-FITC-PI kit (Sigma). This method is based on Vidya Priyadarshini et al. method’s (2010). The cells were resuspended in annexin v binding buffer at a concentration of 0.25-1.0 x 107 cells/ml after being washed twice with cold cell staining buffer. Then, 100 l of the cell suspension was transferred to a 5 ml test tube, along with 5 l of FITC annexin v and 10 l of a 20 g/ml PI (propidium iodide) solution. After that, the cells were vortexed slowly and incubated in the dark for 15 minutes at room temperature (25°C). After that, 400 l of annexin v binding buffer was added to each tube, which was then examined using a flow cytometer using the proper machine settings (44). Cell Pro Quest software was used to examine the data.

### Data collection and data analysis

The software Graphpad Prism 9 and SPSS version 25 were used to analyze the data. The Shapiro Wilk test and the Levene homogeneity test were used to ensure that the data was normal. The apoptosis test and cleaved caspase-3 expression both had a significant p value of 0.05, indicating that the data were not normally distributed. As a result, the statistical test was continued using the Kruskal-wallis test with a statistically significant p value of 0.05. There was also a Spearman correlation test and a Dunn’s multiple comparison *post hoc* test. Meanwhile, the Bcl-2 and BAX expressions revealed a significant p value > 0.05 in the Levene homogeneity test, indicating that the data were not normally distributed. As a result, the statistical test was continued using the one-way ANOVA test with a statistically significant p value of 0.05. This study used the Pearson correlation test and Tukey’s multiple comparison *post hoc* test.

## Result and discussion

There are main mechanisms underlying cervical cancer, namely HPV infection, HPV persistence, progression to dysplasia, and invasion (45). At the molecular level, there is integration of the viral genome into the host genome leading to overexpression of the E6 and E7 proteins of HPV (46). The E7 protein binds and degrades the retinoblastoma gene (*pRb*) (7) resulting in increased E2F activation associated with viral differentiation and replication (4). Meanwhile, E6 forms a trimeric complex with E3 ubiquitin ligase E6AP and DNA-binding domain (*DBD*) *p53* which results in polyubiquitination and degradation of *p53* (7).

One of the most important and most studied cancer signaling pathways is the *p53* tumor suppressor pathway (47). In normal cells, the activity of *p53* is low. However, if there are many stress signals and DNA damage occurs, there will be an increase in *p53* levels which results in the activation and transcription of hundreds of genes with important roles in cell cycle arrest, aging, metabolism, differentiation, and apoptosis. The action of *p53* aims to ensure that abnormal cells fail to proliferate and prevent the accumulation of oncogenic mutations that lead to the development of malignant tumors (48). If the *p53* gene is damaged, then tumor suppression is greatly reduced. The tumor suppressor gene *p53* regulates the expression of Bcl-2 and BAX (9). The two cytoplasmic proteins Bcl-2 and BAX act as inhibitors and promoters of apoptosis (49).

Apoptosis will only occur if a signal is given. There are various conditions that will cause the apoptotic pathway to become active, namely DNA damage or uncontrolled cell proliferation. In cancer, apoptotic activity is delayed. There are many ways cancer cells avoid apoptosis, namely inhibition of caspase function or inactivation of apoptotic triggers. Upregulation of the anti-apoptotic protein Bcl-2 and loss of BAX are the dominant evasion methods. Overexpression of the Bcl-2 protein was detected in more than half of cancer cases. this causes tumor cells to be resistant to any apoptotic stimuli (20, 21).

Currently, the main therapies used to treat cervical cancer are surgery, radiotherapy, and chemotherapy (22). These methods have limitations. Toxicity to normal cells, adverse side effects, and drug resistance are major barriers to chemotherapy use (24). Toxicity limits the usefulness of anticancer agents which is also the reason patients discontinue treatment (24). Anticancer drug resistance is reported as a serious problem and accounts for 90% of chemotherapy failures (25). Because of the current treatment limitations, it is important to explore the development of new anticancer drugs. Natural marine products have been used as compounds for drug discovery (26). Secondary metabolite compounds from marine products are seen as potential sources of active medicinal compounds (27). Seaweed is rich in essential nutrients, minerals and vitamins. It contains polysaccharides, polyphenols, and sterols and has important bioactive properties such as antioxidant, anti-inflammatory, anticancer, antidiabetic, and anticoagulant (50, 51). Sea grapes is a term for varieties of green seaweed of the genus *Caulerpa* (28).

The purpose of this study was to determine the effect of *C. racemosa* extract on apoptosis and viability of *HeLa* cancer cells and their mechanism of action. *HeLa* cells were treated with *C. racemosa* for 24 hours before apoptotic and anti-proliferative activity was observed.

### Pro-apoptotic activity of C. racemosa extract

Measurement of the pro-apoptotic activity of three doses of *C. racemosa* was performed using flow cytometry. *HeLa* cells were treated with four doses of *C. racemosa* and stained using Annexin V (AV) and Propium iodide (PI) as markers of apoptosis. Cells that express AV and PI (AV+/PI+) are cells that enter the late stage of apoptosis, while cells that express only AV (AV+/PI-) are cells that are still in the early stage of apoptosis.

**Figure 2:**
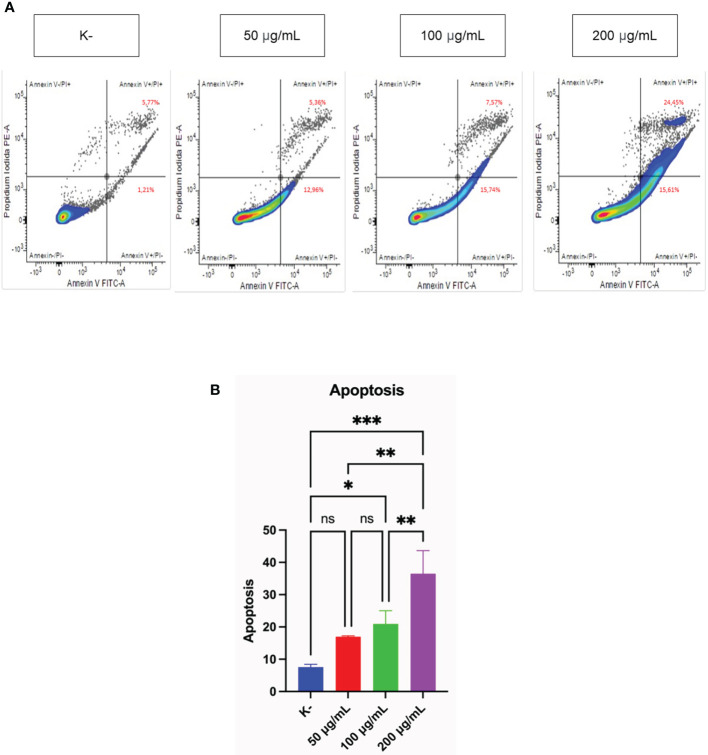
Pro-apoptotic activity of *C. racemosa* extract on *HeLa* cells *in vitro* after 24 hours of treatment. **(A)** Total apoptosis in *HeLa* cells was measured using flowcytometry with Annexin V/PI staining. Annexin V (-)/PI (-): viable cells; Annexin V (+)/PI (+): late apoptosic cells; Annexin V (+)/PI (-): early apoptotic cells; Annexin V (-)/PI(+): necrotic cells. **(B)** Graph of the average total apoptosis, data presented in the form of mean ± standard deviation, *p ≤ 0,05; **p ≤ 0,01; ***p<0,001 (Kruskal-Wallis, Dunn’s multiple comparison *test*). ns, not significant (p>0.05).

*C. racemosa* extract significantly increased total apoptosis in *HeLa* (AV+/PI+) and (AV+/PI-) cells (p < 0.05, Kruskal Wallis test). Along with the increase in dose, total apoptosis in *HeLa* cells also increased significantly, especially at a dose of 200 μg/mL compared with the control group (p < 0.05, Dunn’s *Post Hoc* test). Correlation test showed a perfect positive correlation between dose and total apoptosis in *HeLa* cells (p < 0.05, Spearman test) (**Figure 2**).

Flowcytometry analysis with Annexin V/PI staining was performed to detect whether the cells were viable, apoptotic, or necrotic through differences in plasma membrane integrity and permeability (52). Cells undergoing apoptosis will express phosphatidylserine (PS) on the outer membrane that can bind to Annexin V (53). Propidium iodide (PI) stains cells that have entered the late phase of apoptosis or cell necrosis (52). *HeLa* cancer cells experienced an increase in total apoptosis after administration of *C. racemosa* extract. Polyphenols, an ingredient in seaweed, can be used as anticancer agents by suppressing proliferation, inducing apoptosis and stimulating oxidative stress (54). Polysaccharides obtained from green algae *C. racemosa* showed antitumor activity. When used doses of 50, 100, and 200 mg/kg/day, the levels of inhibition against hepatocellular carcinoma cell lines in rats (H22 cell lines) were 59.5%-83.8% (48 hours) and 53.9% (14 days) in mice with dose of 100 mg/kg/day (55). Research was conducted on six Sri Lankan seaweeds, namely Chondrophycus ceylanicus, Gelidiella acerosa, Gracilaria corticata, Sargassum cassifolium, Chaetomorpha crassa, and *C. racemosa* with doses of 50, 100, and 200 μg/mL. It was found that *C. racemosa* was the species with the best potential to induce apoptosis against HL-60 leukemia cancer cells (26). Therefore, *C. racemosa* extract has the potential to induce apoptosis in cervical cancer cells.

### Mechanism of *C. racemosa* as pro-apoptosis through the expression of cleaved caspase-3

**Figure 3:**
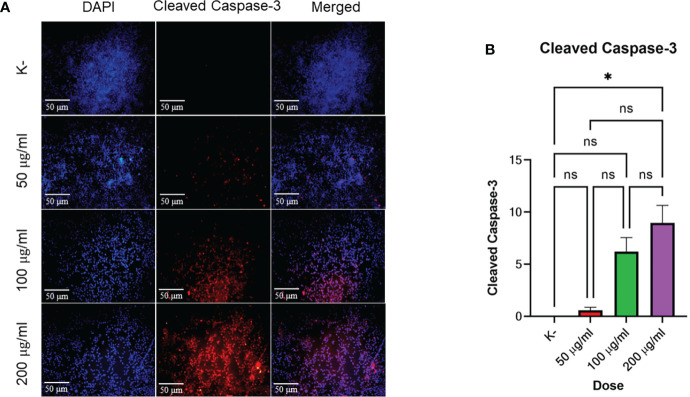
*C. racemosa* induces caspase-3 expression in *HeLa* cells *in vitro* after 24 hours of treatment. **(A)** Expression of cleaved-caspase-3 compared with DAPI on immunofluorescence assay. **(B)** Graph of the mean intensity of cleaved caspase-3. Data are presented in the form of mean ± standard deviation, *p ≤ 0,05 (Kruskal-Wallis, Dunn’s multiple comparison *test*). ns, not significant (p>0.05).

*HeLa* cell apoptosis was identified by administering four doses of *C. racemosa* extract for 24 hours. The doses used were 0 μg/mLas control, 50 μg/mL, 100 μg/mL, and 200 μg/mL. Expression of cleaved caspase-3 was measured using immunofluorescence method with rabbit antibody. The administration of graded doses of *C. racemosa* extract showed an increase in the expression of cleaved caspase-3 at 24 hours of the treatment group compared to the control group (p<0.05) (**Figure 3**).

Based on the data above, incremental dosing increased the expression of cleaved caspase-3 as the dose of *C. racemosa* extract increased. Increased expression of cleaved caspase-3 after incubation for 24 hours proved that *C. racemosa* extract has a pro-apoptotic mechanism against *HeLa* cells of cervical cancer. The results of the Kruskal-Wallis test showed that the independent variable (dose of *C. racemosa* extract) and the dependent variable (cleaved caspase-3) were statistically significant with a p value of 0.018 (p<0.05). Meanwhile, the Spearman correlation test between the dose levels of *C. racemosa* and the increased expression of cleaved caspase-3 had a perfect positive correlation with an R value of 0.957.

HPV E6 protein has oncogenic activity that binds and degrades *p53* which has anti-proliferative and pro-apoptotic activity in response to DNA damage. In the cytoplasm, cytochrome c interacts with APAF 1, procaspase-9, and ATP to form multi-protein complexes called apoptosomes. As a result of this interaction, procaspase-9 is converted into its active form, then acts on procaspase-3 and forms caspase-3. If *p53* is absent or inactivated by mutation, apoptosis does not occur, seI with damaged DNA persists, and may become cancer cell progenitors (13). *C. racemosa* which is rich in metabolites such as phenols, saponins, tannins, flavonoids, xanthoproteins, sesquiterpenoids, diterpenoids, -sitosterol, caulerpin, caulerpicin, caulerpenyne, and epigallo catechins (56) showed maximum effective activity for anti-proliferative activity and Reactive Oxygen Species (ROS) inhibition including transcriptional expression of key cancer and apoptosis-associated genes (56, 57).

### Mechanism of C. racemosa as pro-apoptosis through expression of Bcl-2 and BAX

**Figure 4:**
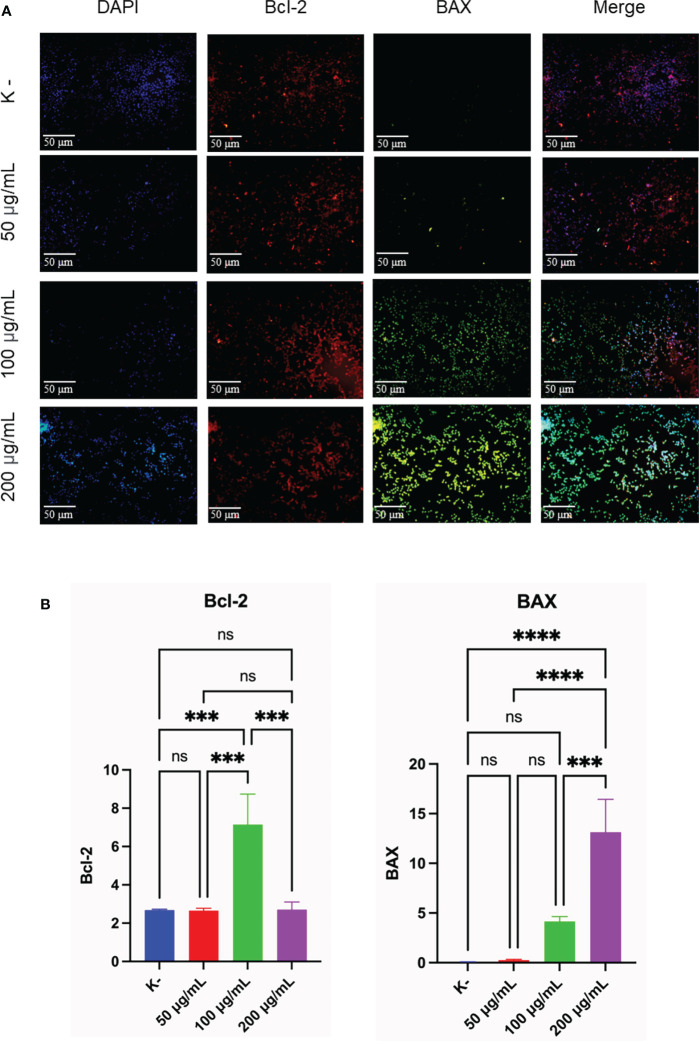
*C. racemosa* induces Bcl-2 and BAX expression in *HeLa* cells *in vitro* after 24 hours of treatment. **(A)** Expression of Bcl-2 and BAX compared with DAPI on immunofluorescence assay. **(B)** Graph of the average intensity of Bcl-2 and Bax. Data is presented in the form of mean ± standard deviation, ***p<0,001; ****p<0,0001 (*One way ANOVA, Tukey’s multiple comparison test*). ns, not significant (p>0.05).

To determine the apoptotic activity, the expression of Bcl-2 and BAX as an indicator of apoptosis was observed. *HeLa* cells were given *C. racemosa* extract with four different doses for 24 hours, 0 μg/mL as control, 50 μg/mL, 100 μg/mL, and 200 μg/mL. All three doses showed an increase in apoptotic activity which was indicated by an increase in the expression of BAX as a proapoptosis (p value < 0.05, One way ANOVA). Based on the data, it was found that as the dose of *C. racemosa* extract increased, BAX expression also increased. There was a significant increase in BAX expression at a dose of 200 μg/mL compared to the control group and a dose of 50 μg/mL at 24 hours after treatment (p value < 0.05 using Tukey’s multiple comparison test). There was a strong positive correlation between dose and BAX expression (p value < 0.05, correlation coefficient 0.880 with Pearson’s test). In addition to the expression of BAX, observations were made on the expression of Bcl-2 as an anti-apoptotic agent. Statistical tests showed significant differences between groups (p value < 0.05, One way ANOVA and Tukey’s multiple comparison test). The increase in Bcl-2 expression is a defense mechanism of *HeLa* cells when *C. racemosa* extract is given. This event was offset by an increase in the expression of BAX as a proapoptosis so that the cells continued to enter the apoptotic phase. In general, all three doses of *C. racemosa* extract modulated apoptotic events in *HeLa* cancer cells compared to the control group (**Figure 4**).

Immunofluorescence staining of Bcl-2 and BAX showed anticancer activity of *C. racemosa* with increased apoptotic activity of *HeLa* cancer cells. Bcl-2 is a major member of the anti-apoptotic Bcl-2 that functions to directly bind and inhibit the proapoptotic Bcl-2 protein (58). BAX is a member of the proapoptotic Bcl-2 family that is dormant in the cytosol (59). BAX is considered to be an executor of mitochondrial outer membrane permeabilization (MOMP) during apoptosis (60). Activated BAX will translocate to MOM and subsequently assemble a protein complex called permeability transition pore (PTP) to create an opening through both mitochondrial membranes, eventually causing MOM to rupture due to swelling of the mitochondrial matrix (61). The MOMP process causes the release of multiple mitochondrial apoptogenic proteins into the cytosol to trigger the apoptotic cascade (60).

*C. racemosa* extract significantly increased the proapoptotic BAX expression. The increased expression of Bcl-2 is a response of cell defense to the administration of *C. racemosa* extract. However, in the end *HeLa* cells still enter the apoptotic phase. *Caulerpa* has bioactive metabolites namely alkaloids, terpenoids, flavonoids, steroids and tannins and its bioactivity has been reported against many diseases including cancer (54). Flavonoids are reported to support the induction of apoptosis by modulating Bcl-2 which is an anti-apoptotic factor. Bcl-2 functions by preventing mitochondrial pore formation and cytochrome release thereby inhibiting the initiation of apoptosis (62). Research by Bhakti et al. in 2020, used *Caulerpa* spp at a dose of 20-500 g/mL. It was found that *Caulerpa* spp has the potential to increase *p53* gene expression in *HeLa* cells (57). The expression of BAX which is also a regulator of apoptosis is increased. Meanwhile, the expression of the *CDC2* gene, which plays a role in tumor formation, was doubled by *C. racemosa* extract (57).

### C. racemosa extract activity in reducing HeLa cell viability

**Figure 5:**
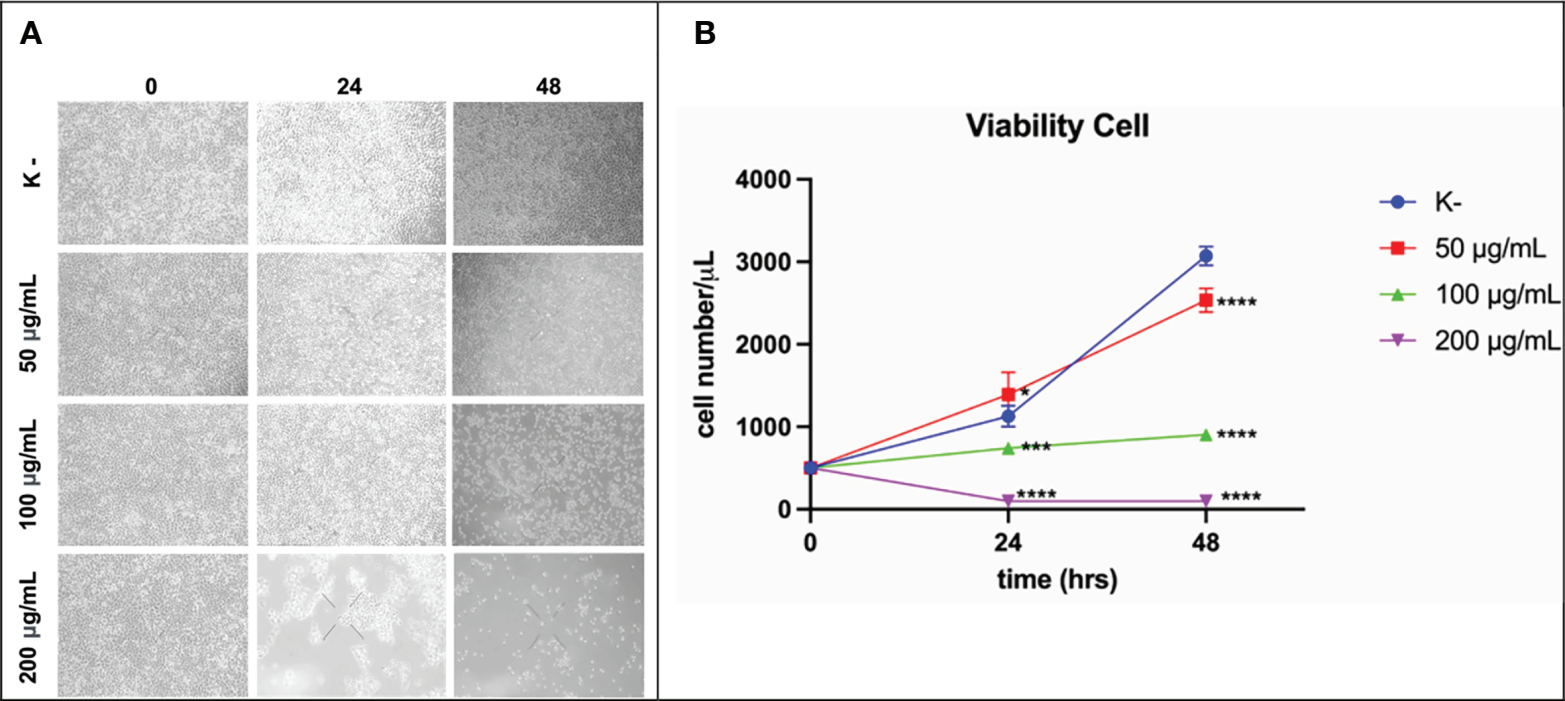
Activity of *C*. *racemosa* extract in reducing *HeLa* cell viability *in vitro* after 24 and 48 hours of treatment. **(A)** Microscopic viability of *HeLa* cells. **(B)** Graph of decreased viability of *HeLa* cells at 24 and 48 hours, *p ≤ 0,05; ***p<0,001; ****p<0,0001 (*Kruskal-Wallis, Dunn’s multiple comparison test*).

*HeLa* cell viability was measured at 24 h and 48 h. *HeLa* cells were given four doses of *C. racemosa* extract (0 μg/mL, 50 μg/mL, 100 μg/mL, 200 μg/mL). *HeLa* cell viability was measured using the trypan blue exclusion assay. After 24 hours of treatment, *C. racemosa* showed anti-proliferative activity indicated by a decrease in the number of viable cells compared to the control group, especially at a dose of 50 μg/mL compared to a dose of 200 μg/mL (p < 0.05, Kruskal Wallis test and Dunn’s *Post Hoc* test)). At 48 hours after treatment, anti-proliferative activity of *C. racemosa* was also found, especially at a dose of 200 μg/mL compared to the control (p < 0.05, Kruskal Wallis test and Dunn’s *Post Hoc* test). There is a perfect negative correlation between the dose of *C. racemosa* and the viability of *HeLa* cells, which means that the larger the dose used, the fewer the number of *HeLa* cells that appear viable on the trypan blue exclusion assay (p < 0.05, *Spearman test*) (**Figure 5**).

The antiproliferative activity of *C. racemosa* was evidenced by a significant decrease in the viability of *HeLa* cancer cells after 24 and 48 hours of treatment. *Caulerpa*’s anticancer metabolites such as caulerpenyne (Cyn), caulerpin, caulersin, and racemosin C, have unique structural features and are known to exhibit different effects on cancer cells. These metabolites are reported to influence microtubule dynamics, unfolded protein response, mitochondrial health, cell cycle development, metabolic pathways and stress (35). Caulerpenyne alters ATP-dependent Ca2+ storage in intracellular organelles, protein phosphorylation, and DNA synthesis. Cyn induces the inhibition of SK-N-SH cell line neuroblastoma proliferation by inducing tubulin aggregation which is responsible for the inhibition of microtubule polymerization. In another study by Fischel et al, Cyn inhibited growth in eight human cancer lines by causing an initial shift of cells to the S phase followed by blocking in the G2/M phase (63). *C. racemosa* affects the cell cycle resulting in inhibition of the proliferation of *HeLa* cancer cells.

Of course, taking into account the effective doses obtained from this study, it is hoped that there will be further studies. Further studies that need to be carried out are tests on the ability of *C. racemosa* extract to inhibit Hela cells from penetrating the extracellular matrix so that it is certain that the administration of the extract can inhibit the metastasis process of cancer cells. Moreover, trials on model animals or preclinical trials are also a concern for our future research to observe their toxicity.

## Conclusion and practical implications

This study showed the antiproliferative activity of *C. racemosa* which was characterized by a decrease in cell viability and the effect of increasing apoptotic activity on *HeLa* cells, which are cervical cancer cells. *C. racemosa* extract with various concentrations significantly increased the expression of pro-apoptotic protein BAX, cleaved caspase-3, total apoptosis, and decreased *HeLa* cell viability. The results of the study indicate that *C. racemosa* has potential promising as an anti-cancer that can be studied and observed further.

## Data availability statement

The raw data supporting the conclusions of this article will be made available by the authors, without undue reservation.

## Author contributions

HP, NT, and FM: conduct experiments, analyzed data, write manuscript, design research, and conceptualize ideas; while FN, MY, DW, SB, VD, and AS contribute to data analysis, critiquing manuscript, interpreting manuscript results, assisting in the processing of data, as well as helping to revise and graphical abstract editing. All authors have read and also approved this final manuscript.

## Funding

The study was conducted with the grant from ‘Pelaksanaan Program Riset Keilmuan untuk perguruan Tinggi tahun Anggaran 2021 - Institute of Research and Community Services Brawijaya University (LPMM) Brawijaya University with number 016/E4.1AK.RA/2021.

## Acknowledgments

The authors thank all of the contributors for their outstanding help in research and also in formatting the paper.

## Conflict of interest

The authors declare that the research was conducted in the absence of any commercial or financial relationships that could be construed as a potential conflict of interest.

## Publisher’s note

All claims expressed in this article are solely those of the authors and do not necessarily represent those of their affiliated organizations, or those of the publisher, the editors and the reviewers. Any product that may be evaluated in this article, or claim that may be made by its manufacturer, is not guaranteed or endorsed by the publisher.
